# Genome-wide host-associated population structure of *Sarcoptes scabiei*: One Health implications

**DOI:** 10.1016/j.soh.2026.100168

**Published:** 2026-09-03

**Authors:** Fang Fang, Pasi K. Korhonen, Siyi Huang, Barbara Moroni, Luca Rossi, Anna R. Molinar Min, Zhijun Hou, Vito Colella, Qingyou Liu, Jacques Guillot, Robin B. Gasser

**Affiliations:** a Department of Parasitology, College of Animal Science and Technology, Guangxi University, Nanning, China; b Department of Veterinary Biosciences, Melbourne Veterinary School, Faculty of Science, The University of Melbourne, Parkville, Victoria, Australia; c Istituto Zooprofilattico Sperimentale del Piemonte, Liguria e Valle d'Aosta, Italy; d Department of Veterinary Sciences, University of Turin, Turin, Italy; e College of Wildlife and Protected Area, Northeast Forestry University, Harbin, China; f College of Animal Science, South China Agricultural University, Guangzhou, China; g Department of Dermatology, Parasitology, Mycology, Oniris, Nantes, France; h Univ Angers, Univ Brest, IRF, SRF, Angers, France

**Keywords:** One Health, Scabies, Sarcoptic mange, *Sarcoptes scabiei*, Host-associated populations, Genomics

## Abstract

**Background:**

Scabies, caused by the mite *Sarcoptes scabiei*, affects more than 200 million people at any one time and can be complicated by severe secondary bacterial disease. The same mite causes sarcoptic mange in companion animals, livestock and wildlife. Population-scale nuclear genomic data spanning diverse hosts and geographical regions remain limited.

**Methods:**

We generated whole-genome sequence data for 106 *S. scabiei* samples and incorporated 19 publicly available datasets from humans, companion animals, livestock and wildlife. After quality filtering, 83 of 125 sample-level datasets were retained. We assessed ancestry, phylogenetic relationships, nucleotide diversity, genetic differentiation, gene-level variation and candidate selection signals.

**Results:**

We identified 1,568,031 filter-passing nuclear variants, including 698,136 single-nucleotide polymorphisms; sample-level heterozygosity estimates ranged from 0.27% to 11%. ADMIXTURE, principal component and phylogenetic analyses supported seven ancestry clusters broadly corresponding to host-associated groups, with pairwise fixation-index estimates ranging from 0.22 to 0.92. Multi-metric analysis identified 1600 candidate selection blocks – eight hard-sweep, 486 soft-sweep and 1106 ancient-adaptation blocks – and six conserved low-diversity blocks. Variable genes showed nominal enrichment for DNA repair, cell adhesion and signalling, whereas invariable genes showed nominal enrichment for ribosomal function, oxidative phosphorylation and mitochondrial processes.

**Conclusions:**

This expanded multi-host analysis reinforces and extends evidence that host association is a prominent correlate of genome-wide population structure in *S. scabiei*. The findings provide a population-genomic resource for future host-stratified One Health surveillance and investigations of transmission, adaptation and acaricide resistance in scabies and sarcoptic mange.

## Graphical abstract

**Figure:**
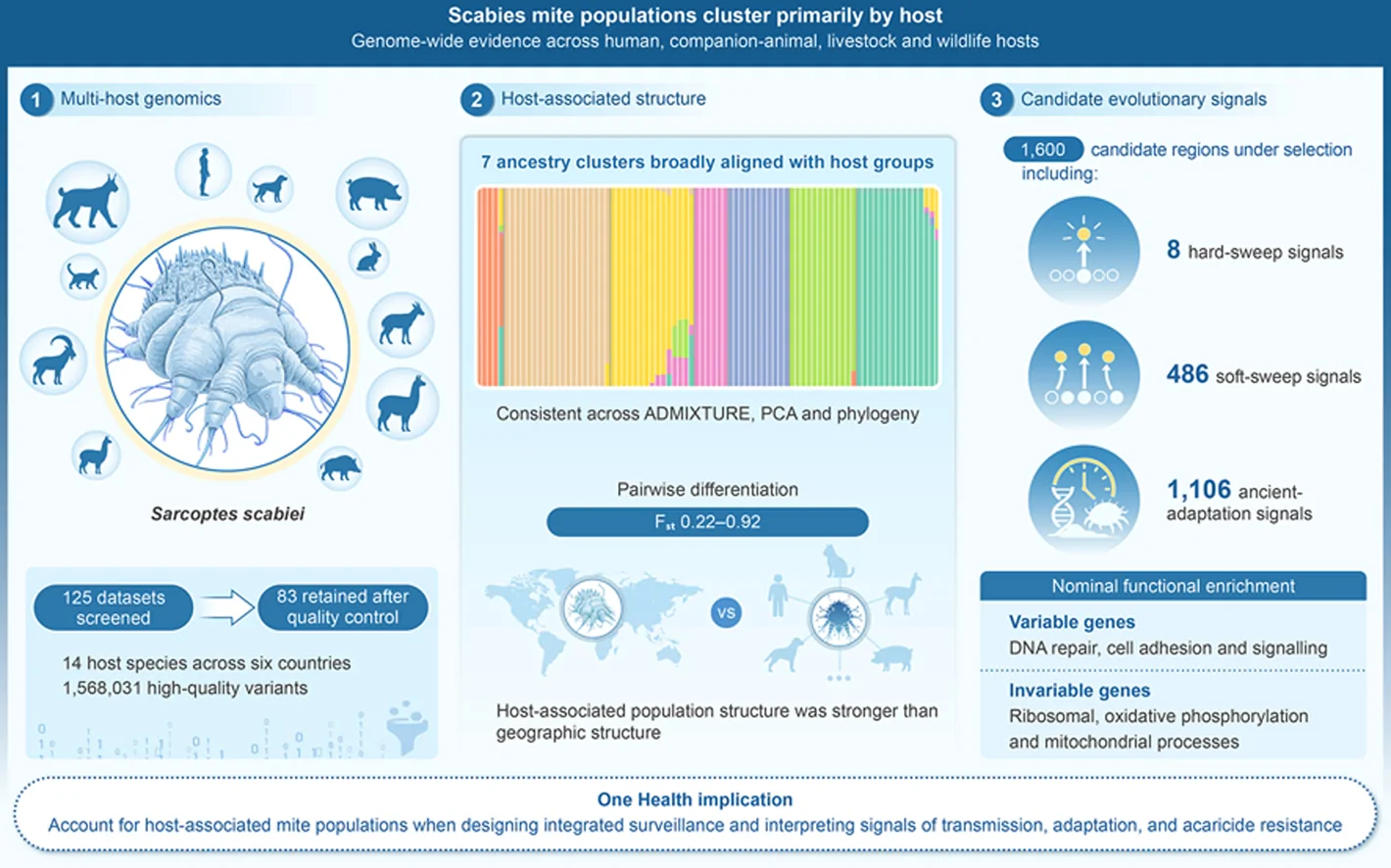

## 1 Introduction

Scabies is a contagious parasitic skin disease of humans caused by the mite *Sarcoptes scabiei*. Globally, it remains one of the commonest dermatological conditions, with more than 200 million people estimated to be affected at any one time, particularly in disadvantaged and resource-limited communities [[1], [2], [3]]. The clinical manifestations of scabies result from mite infestation of the epidermis and include intense pruritus, papular rash and, in severe cases, crusted lesions. These lesions are frequently complicated by secondary bacterial infections with *Streptococcus pyogenes* and/or *Staphylococcus aureus*, which can progress to life-threatening sequelae such as sepsis, acute post-streptococcal glomerulonephritis and rheumatic heart disease [4]. Accordingly, the burden of scabies extends well beyond dermatological disease, contributing substantially to morbidity in endemic communities. Treatment and control are further constrained by the costs of therapy, the limited availability of acaricides and the logistical challenges of mass drug administration in resource-poor settings [5]. Reflecting this substantial global burden, the World Health Organization has included scabies in its roadmap for neglected tropical diseases for 2021–2030 [1]. The significance of *S. scabiei* also extends beyond human health, as the same mite causes sarcoptic mange in companion animals, livestock and wildlife [6]. Mange outbreaks in wild species, including foxes, wombats and ungulates, have been linked to marked population declines and, in some instances, local extinctions [7]. In livestock, chronic sarcoptic mange can impair productivity and compromise animal welfare [6]. In dogs, mange is a common and debilitating condition worldwide, and occasional transmission to humans may occur following close contact [6]. This zoonotic potential, combined with the broad host range of *S. scabiei*, underscores the importance of the parasite within a One Health context.

Despite its widespread distribution and considerable burden, many aspects of the biology, epidemiology and host–parasite interactions of *S. scabiei* remain poorly understood. Historically, genetic studies of this mite relied primarily on mitochondrial DNA and microsatellite markers [[8], [9], [10], [11]]. These early investigations provided some evidence of host-associated clustering and raised the possibility of separate transmission cycles. Microsatellite analyses showed that dog-derived and human-derived mites were genetically distinct, supporting the concept of host-associated lineages and helping to explain differences in clinical epidemiology [9]. Nevertheless, the genetic markers used at the time lacked the resolution to determine the extent of gene flow among host-associated populations, evaluate evidence for cross-species transmission, characterise genome-wide differentiation or identify genomic regions shaped by selection. Consequently, key aspects of host-associated population structure and evolutionary divergence remain unresolved. Resolving these patterns is particularly important, given the exceptional host range of *S. scabiei* and its ability to persist in diverse ecological contexts [6,7,12].

Advances in genomic technologies have begun to address these knowledge gaps. Korhonen et al. [13] reported a high-quality nuclear reference genome for *S. scabiei*, representing a major advance in mite genomics. This assembly, generated using a combination of short- and long-read sequencing, provided a foundation for the annotation of gene families of functional and therapeutic relevance, including G protein-coupled receptors, ion channels, kinases, proteases, protease inhibitors and allergens. These gene sets are directly relevant to understanding mite physiology, host immune interactions and the mechanisms of action of acaricides. The reference genome also provided initial insights into the contraction of detoxification gene families, which may contribute to susceptibility to commonly used acaricides. A comparative genomic analysis of astigmatic mites, including *S. scabiei*, identified gene-family changes associated with parasitism [14]. Xu et al. [15] further revealed strong population structuring by host species and selective sweeps in gene families linked to immune modulation, apoptosis and proteolysis, and reconstructed the demographic history of *S. scabiei* populations, inferring divergence times and patterns of host adaptation. Together, these studies provide complementary genomic resources and evolutionary insights. However, population-scale genomic analyses encompassing diverse host species and geographical regions remain limited.

To address this gap, we build on the four-host comparison of Xu et al. [15] by analysing 83 quality-filtered whole-genome datasets from humans, companion animals, livestock and diverse wildlife hosts across multiple geographical regions. We use an integrated population-genomic framework to characterise genome-wide variation, host- and geography-associated population structure, and signatures of selection and evolutionary constraint. This expanded multi-host analysis provides a basis for investigating variation among *S. scabiei* populations and for informing future host-stratified One Health surveillance of scabies and sarcoptic mange.

## 2 Methods

### 2.1 Short-read sequencing of genomic DNA samples

Genomic DNA samples of *S. scabiei* from humans, companion animals (cats and dogs), domestic livestock (pigs and rabbits) and wildlife (including Eurasian lynx, Himalayan goral, guanaco, Iberian ibex, Iberian lynx, llama, vicuña, wild boar and wild rabbit) (Table S1) were sourced from previous investigations and new field collections [16,17]. DNA was extracted from individual mites or from pools of three to 30 mites (Table S1), depending on the amount of parasite material available, using the QIAamp DNA Micro Kit (Qiagen) following established protocols [18]. For 19 publicly available pooled datasets, the exact number of mites contributing to each pool was not reported in the source metadata (Table S1).

Following quality control, 10 ng of genomic DNA was subjected to enzymatic shearing, end-repair and A-tailing. Indexed adaptors were ligated to both ends of DNA fragments, after which libraries were amplified by polymerase chain reaction (PCR) and circularised to produce single-stranded circular DNA molecules. These were subsequently amplified by rolling circle amplification to generate DNA nanoballs, which were loaded on to flow-cells and sequenced using the DNBSEQ platform (BGI, Shenzhen), yielding 150 bp paired-end reads. For some samples representing individual mites and containing limited amounts of genomic DNA, multiple sequencing runs were conducted, and the resulting sequence data were combined. In total, sequence data were generated from 106 samples (Table S1).

### 2.2 Recording nucleotide variation

Raw FASTQ data were quality-filtered using SOAPnuke v1.5.6 (BGI) [19]. High-quality read pairs were mapped to the *S. scabiei* reference genome [15] using the *Escalibur* pipeline [20], which incorporates the Burrows–Wheeler Aligner (BWA-MEM v0.7.12) [21] and duplicate marking with Picard v2.23.3 (https://broadinstitute.github.io/picard). To identify and remove host-derived sequences, read sets were also aligned to the corresponding host genome, and host-derived reads were excluded.

Single-nucleotide polymorphisms (SNPs) and insertion/deletion events (indels) were identified using the Genome Analysis Toolkit (GATK v4.1.3.0) [22]. Variants were called using HaplotypeCaller in genomic Variant Call Format (gVCF) mode; gVCFs were combined using CombineGVCFs and jointly genotyped using GenotypeGVCFs, followed by initial filtering using VariantFiltration and vcftools v0.1.17 [23]. Variant calls were normalised using bcftools v1.6, and a quality- and frequency-filtered variant set was retained after excluding sites that met any of the following criteria: quality by depth (QD) < 2.0, Fisher strand bias >60.0, mapping quality (MQ) < 40.0, mapping-quality rank-sum test (MQRankSum) < −12.5, read-position rank-sum test (ReadPosRankSum) < −8.0, Phred-scaled variant quality score (QUAL) < 30, read depth (DP) < 5, missingness ≥65%, or minor allele frequency < 0.05. Samples with >30% missing data or heterozygosity > 0.11 were removed, whereas a higher heterozygosity threshold (>0.3) was applied to pooled-mite samples. Per-sample missingness was calculated using plink2 v2.0.0. Genotype quality filters were applied to single-mite datasets but not to pooled-mite datasets, because non-diploid allele proportions in pooled samples can yield low genotype-confidence scores despite reliable site-level support, potentially leading to the systematic exclusion of informative sites.

For pooled-mite samples, standard-mode GATK calls were cross-validated against SNVer [24], a variant-calling tool designed for pooled sequencing data. Overlapping variant calls were identified, and Pearson correlation coefficients were calculated between GATK-derived discrete genotype-frequency estimates and SNVer-derived continuous allele-frequency estimates. For pooled-mite datasets, allele-frequency and heterozygosity values were treated as composite sample-level estimates rather than individual-mite values. The analysis initially comprised 125 sample-level whole-genome datasets: 106 generated in this study and 19 publicly available datasets (Table S1). After quality filtering, 83 datasets were retained and 42 were excluded from downstream analyses. Dataset identifiers, quality-control outcomes and reasons for exclusion are provided in Table S1. Invariant sites, defined as sites at which all samples carried the reference allele, were extracted and filtered for depth (1 ≤ DP ≤ 500) and missingness (< 0.95).

### 2.3 Analyses of population structure

Population structure was investigated using multiple complementary approaches. Ancestry clusters were estimated iteratively using ADMIXTURE v1.3.0 [25] for cluster counts from 2 to 12, with five-fold cross-validation. The cluster count with the lowest cross-validation error was selected, and the resulting clusters were used to define host-associated clusters for subsequent analyses.

Genome-wide diversity and differentiation statistics were estimated using pixy v2.0.0 [26,27] in 50-kb genomic windows. Within-cluster variation was characterised using nucleotide diversity (*π*), Watterson's theta (*θ*_W_) and Tajima's *D*, whereas between-cluster differentiation and absolute divergence were estimated using fixation index (*F*_ST_) and absolute nucleotide divergence (*D*_XY_), respectively. To incorporate invariant information and account for missing data, *π*, *θ*_W_, Tajima's *D* and *D*_XY_ were estimated from callable, filter-passing variant and called invariant nucleotide positions, rather than from variant positions alone. The called invariant records represented individual nucleotide positions, with one Variant Call Format (VCF) record per site, rather than compressed gVCF reference blocks. The pixy input was therefore a standard VCF containing both variant and invariant records, and the --gvcf option was not used. The same filtered set of nucleotide positions was analysed for all clusters. Nucleotide-diversity estimates were reported as proportions, representing average pairwise nucleotide differences per analysed nucleotide position. *F*_ST_ was estimated from filter-passing SNPs within the corresponding windows. The resulting window-specific estimates were visualised across the nine nuclear chromosomes of *S. scabiei* [15] using pycirclize v1.10.1.

Principal component analysis (PCA) was conducted using scikit-learn v1.8.0 [28]. SNPs were pruned for linkage disequilibrium using a squared correlation coefficient threshold of *r*^2^ < 0.2 with SNPRelate v1.44.0 [29]. Using this pruned SNP set, phylogenetic inference was conducted using BEAST2 v2.7.7 [30] under a general time-reversible substitution model, with Markov chain Monte Carlo analyses run for 10 million generations and a burn-in of 20%. Five independent runs were combined using LogCombiner of BEAST2, and consensus trees were produced using TreeAnnotator of BEAST2 and visualised using toytree v3.0.11 [31]. To assess the sensitivity of the inferred population structure to the inclusion of pooled-mite datasets, ADMIXTURE and PCA were repeated after excluding all pooled datasets (R1–R3 and B1–B19).

### 2.4 Gene-level nucleotide diversity and functional annotation

Read coverage and depth across annotated protein-coding genes [15] were calculated using mosdepth v0.3.3 [32]. Genes were retained if ≥ 95% of their length had coverage ≥5 × and mean coverage ≥20 × across all samples. Multicopy genes were identified using OrthoMCL v2.0.4 [33] and excluded.

Per-gene nucleotide diversity was estimated as *π* = *n*/(*n* − 1) × (1 − ∑*p*_i_^2^), where *n* is the total number of alleles sampled at a site and *p*_i_ is the frequency of allele i. For genes containing multiple variant sites, gene-level *π* was calculated by summing site-specific *π* values across each gene and dividing by the corresponding sequence length. Because strict site-level callability filtering left too few validated sites for many loci, the denominator was defined as the full annotated nucleotide length of each gene feature, with all sites within the feature treated as callable. Genes were ranked according to mean *π* and standard deviation and classified as variable or invariable using the upper and lower 10% of the distribution, respectively. Analyses were conducted using both coding sequences (CDS) and full gene models – including introns (Table S2).

Functional annotation was performed by assigning homology-based Kyoto Encyclopedia of Genes and Genomes (KEGG) Orthology (KO) terms [34] from the KOfam database v2026-02-01 using exec_annotation v1.3.0 from the KofamScan package [35], with E-values < 1 × 10^−8^. KO terms were then mapped to KEGG pathways and KEGG BRITE orthologous protein families using the KEGG Orthology Based Annotation System (KOBAS) database [36]. Enrichment of KEGG pathways and BRITE hierarchies was assessed using Fisher's exact test, with significance thresholds of *P* < 0.05 and *P* < 0.01.

### 2.5 Selective sweep and candidate-gene analyses

Clusters defined by ADMIXTURE analysis were used to establish a baseline for identifying candidate selection signatures. Within-cluster metrics (*π*, *θ*_W_ and Tajima's *D*) and *D*_XY_ were estimated from callable, filter-passing variant and called invariant nucleotide positions, whereas *F*_ST_ was estimated from filter-passing SNPs, as described in subsection 2.3. Analyses were performed in non-overlapping 10 kb windows; candidate windows were evaluated using corresponding 50 kb contextual windows.

A hierarchical window-based approach was employed to identify candidate genomic signals of selection while minimising false positives associated with stochastic variation or sequencing gaps. Candidate 10 kb windows were initially identified when *π* fell below the 15th percentile of the genome-wide distribution for a given cluster. Each candidate window was then evaluated using the corresponding 50 kb contextual window and retained only if that contextual window fell below the 30th percentile. Adjacent retained 10 kb windows were merged into contiguous genomic blocks, which constituted the final units used for classification.

To identify candidate signals of host-associated adaptation, a standardised contrast score (Welch's *Z*-score) was calculated as *Z* = (*π*_others_ − *π*_target_)/√((*σ*^2^_target_/*N*_target_) + (*σ*^2^_others_/*N*_others_)), where *π* represents nucleotide diversity, *σ*^2^ the variance and *N* the number of samples; *π*_others_ represents the combined nucleotide-diversity estimate across all non-target clusters. This formulation incorporates both variance and sample size, ensuring that statistical support reflects the underlying data structure.

Final genomic blocks were classified into four categories based on a multi-metric consensus. Candidate hard-sweep blocks were defined by extreme diversity loss (below the 5th percentile), high contrast (*Z* > 4.0) and a strongly negative site-frequency spectrum (Tajima's *D* < −1.0), patterns consistent with relatively recent allele-frequency shifts. Candidate ancient-adaptation blocks were defined by high relative differentiation (*F*_ST_ > 0.4) accompanied by high absolute divergence (*D*_XY_ above the genome-wide median), a pattern consistent with longer-term population separation. Candidate soft-sweep blocks were defined by moderate contrast (*Z* > 2.0) without extreme spectral skew and were interpreted as consistent with selection acting on standing variation. Conserved low-diversity blocks were defined as genome-wide valleys of low diversity (below the 5th percentile across all clusters) with low contrast (*Z* ≈ 0), consistent with strong purifying selection.

Variants associated with these blocks were identified using the absolute allele-frequency difference (ΔAF), calculated as the absolute difference between the estimated allele frequency in the target cluster and the pooled estimate across all other clusters (ΔAF = |AF_target_ − AF_others_|). Variants with ΔAF > 0.8 were classified as highly differentiated, and those with ΔAF >0.95 were considered to show near-complete cluster-level allele-frequency differentiation. Because pooled-mite datasets were included, these quantities represent sample- and cluster-level estimates rather than evidence of fixation in individual mites. Variants were further prioritised using a composite ranking score defined as R_C_ = ΔAF × *Z*.

To rank candidate genes, an importance score was calculated as score = (*Z* × 2) + (I_FS_ × 10) + (*V*_exon_ × 3) + (ΔAF_max_ × 5), where *Z* represents the standardised selection contrast, I_FS_ is an indicator of the presence of a putative exonic frameshift mutation, *V*_exon_ is the number of exonic variants meeting the predefined high-differentiation criterion, and ΔAF_max_ is the maximum estimated allele-frequency difference within the transcript. Genes were classified into three tiers: Tier 1 (score > 25), representing high-priority candidates; Tier 2 (score 15–25), representing intermediate-priority candidates; and Tier 3 (score < 15), representing lower-priority candidates. Within each cluster, genes were ranked first by tier and then by score to prioritise those with the strongest combined evidence. High-confidence variants were further defined using hierarchical criteria, including association with Tier 1 genes, predicted functional impact, near-complete cluster-level allele-frequency differentiation (ΔAF > 0.95), and enrichment of exonic variants within genes. Selection patterns were visualised using Circos and Manhattan plots generated with pycirclize v1.10.1 (https://github.com/moshi4/pyCirclize) and seaborn v0.13.2 [37].

## 3 Results

### 3.1 Genome sequencing and genome-wide variation

Whole-genome sequencing of 106 *S. scabiei* samples from humans, companion animals (cats and dogs), livestock (pigs and rabbits) and wildlife yielded between 387 Mb and 50.6 Gb of high-quality paired-end data per quality-controlled dataset (Table S1). Nineteen publicly available datasets (B1–B19) were also included (Table S1), giving 125 sample-level datasets for initial analysis. After quality filtering and removal of host-derived reads, 88–97% of the remaining reads per dataset mapped uniquely to the *S. scabiei* nuclear reference genome [15]. The number of uniquely mapped reads ranged from 2.03 to 96.95 million per dataset, corresponding to mean genome-wide sequencing depths of 4.85 × to 182 ×. Across the 83 datasets retained after stringent filtering, 1,826,473 filter-passing genomic records were identified, comprising 1,568,031 variant records – 698,136 SNPs, 749,092 indels, 2236 multi-nucleotide polymorphisms (MNPs) and 118,567 complex variants – and 258,442 called invariant records. Sample-level heterozygosity estimates ranged from 0.27% to 11%. A total of 46,545 cluster-specific variant records showed high allele-frequency differentiation (ΔAF > 0.8): 28,794 SNP records, 17,367 indel records and 384 MNP records.

### 3.2 Host-associated population structure and sensitivity analyses

ADMIXTURE analysis identified seven ancestry clusters broadly corresponding to host-associated groups, indicating that genetic structure was more closely aligned with host association than with geographical distribution across the sampled populations: K1 (humans and pigs; Hominidae/Suidae), K2 (wild Caprinae; Bovidae), K3 (domesticated rabbits; Leporidae), K4 (lynxes; Felidae), K5 (wild rabbits; Leporidae), K6 (cats and dogs; Felidae/Canidae), and K7 (guanacos, llamas and vicuñas; Camelidae) (Table 1; Fig. 1A). Genome-wide nucleotide-diversity estimates (*π*), reported as proportions, varied among these clusters. The highest mean estimates were observed in K1 (*π* = 0.1945) and K6 (*π* = 0.1537), whereas lower estimates were observed in K4 (*π* = 0.0126) and K5 (*π* = 0.0225) (Table 2). PCA showed a similar pattern, with the first two components (PC1 and PC2) explaining 43% of total genetic variation and separating host-associated clusters with minimal overlap (Fig. 1B). Subsequent phylogenetic analysis based on unlinked SNPs showed clustering patterns consistent with these ancestry assignments (Fig. 1C), with sample-level datasets grouping according to host association. Human-derived mites from Australia and Italy clustered together irrespective of geographical origin, dog-associated mites from China and Spain formed a separate cluster, and pig-associated mites from Australia, China and Italy grouped together. Wildlife-derived mites, including those from vicuña, llama, ibex, lynx and wild rabbits, formed distinct host-associated clusters, with posterior probabilities of 1.0 for all major nodes.

**Table 1:** Ancestry-cluster characteristics of *Sarcoptes scabiei* inferred using ADMIXTURE. Clusters K1–K7 represent ancestry groups identified by model-based estimation. The number of sample-level datasets (*n*) indicates the number of quality-filtered datasets assigned to each cluster; each dataset represents either an individual mite or a pool of mites. The column labelled ‘Hosts’ lists the principal host groups represented within each cluster, with taxonomic families in parentheses. Purity (%) denotes the percentage of datasets assigned predominantly to the corresponding cluster. Mean ancestry proportion is the mean maximum membership coefficient across the datasets assigned to each cluster.

Cluster	No. of sample-level datasets	Hosts	Purity (%)	Mean ancestry proportion
K1	15	Humans and pigs (Hominidae/Suidae)	100	0.9586
K2	5	Gorals and ibex (Bovidae)	100	0.9999
K3	11	Domesticated rabbits (Leporidae)	100	0.9999
K4	6	Lynx (Felidae)	100	0.9999
K5	12	Wild rabbits (Leporidae)	100	0.9925
K6	15	Cats and dogs (Felidae/Canidae)	100	0.8709
K7	19	Guanacos, llamas and vicuñas (Camelidae)	100	0.9934

**Fig. 1:**
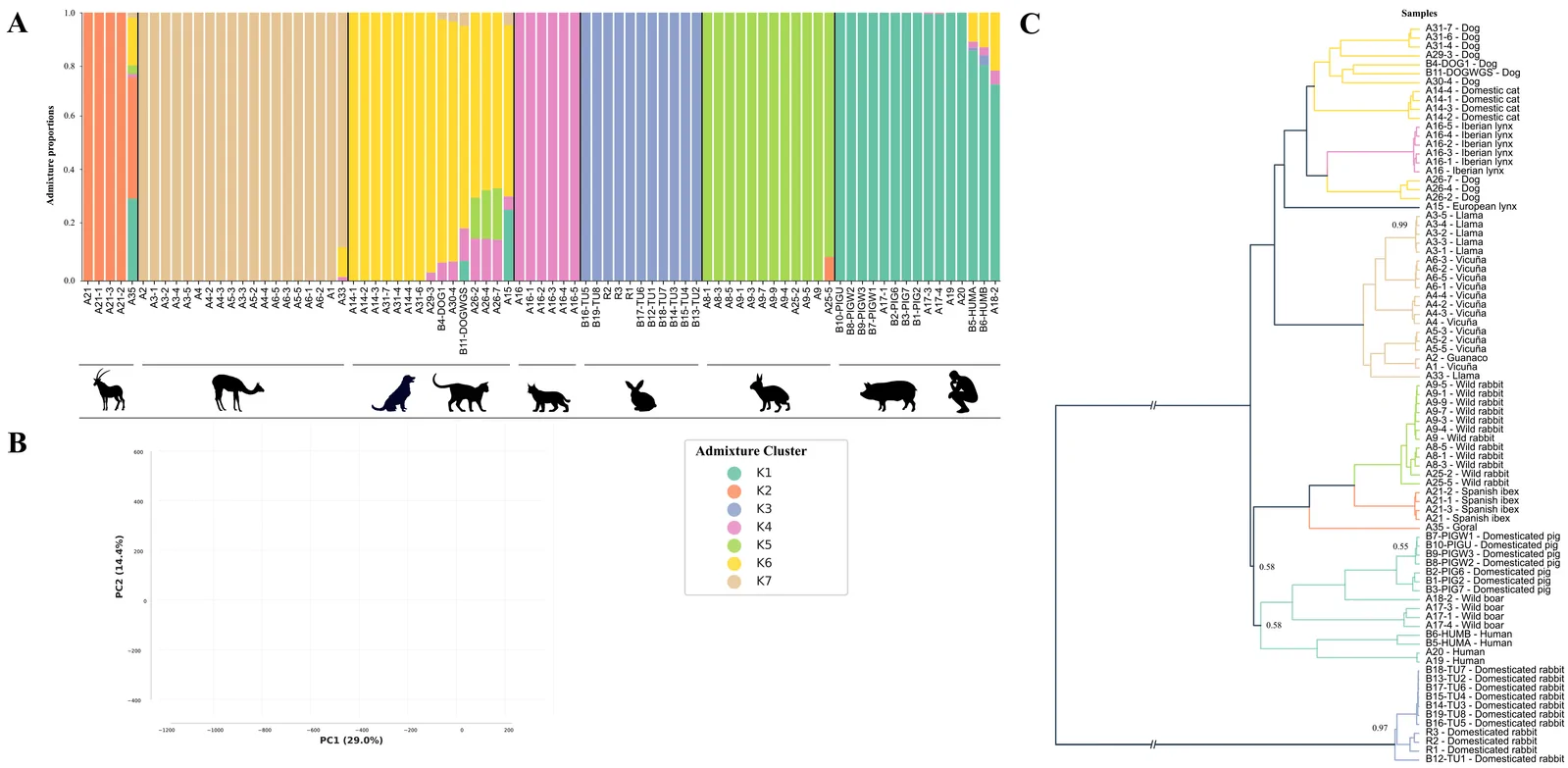
Population structure of *Sarcoptes scabiei* inferred from genome-wide nuclear single-nucleotide polymorphism (SNP) data. (A) ADMIXTURE analysis showing estimated ancestry proportions for 83 quality-filtered sample-level datasets from diverse host species (Table S1), assigned to seven ancestry clusters (K1–K7). Each vertical bar represents one sample-level dataset derived from either an individual mite or a pool of mites; colours indicate the estimated proportion of ancestry assigned to each cluster. (B) Principal component analysis (PCA) of genome-wide SNP data. Each point represents one sample-level dataset and is coloured according to its ADMIXTURE cluster assignment. The first and second principal components (PC1 and PC2) explain 29.0% and 14.4% of the total genetic variance, respectively. (C) Phylogenetic relationships inferred from high-quality, linkage-pruned nuclear SNPs. Each terminal tip represents one sample-level dataset. Branches are coloured according to ADMIXTURE cluster assignment, and node labels indicate posterior probabilities; values of 1.0 are not shown. Across the sampled populations, datasets clustered more closely according to host-associated group than geographical origin.

**Table 2:** Genome-wide nucleotide-diversity (*π*) estimates for ADMIXTURE-defined *Sarcoptes scabiei* clusters K1–K7. The number of sample-level datasets (*n*) indicates the number of quality-filtered datasets contributing to each cluster, including individual- and pooled-mite datasets. Mean, median and standard deviation (SD) summarise the distribution of window-specific cluster-level *π* estimates across 50-kb genomic windows. Values are reported as proportions representing average pairwise nucleotide differences per analysed nucleotide position. All clusters were analysed using the same set of filter-passing variant and invariant nucleotide positions.

Cluster	No. of sample-level datasets	*π* (mean)	*π* (median)	*π* (SD)
K1	15	0.1945	0.1955	0.0429
K2	5	0.0674	0.0650	0.0272
K3	11	0.0706	0.0605	0.0567
K4	6	0.0126	0.0043	0.0255
K5	12	0.0225	0.0073	0.0352
K6	15	0.1537	0.1574	0.0436
K7	19	0.0795	0.0728	0.0495

Pooled-mite datasets were evaluated using two complementary approaches. First, standard-mode GATK calls were cross-validated against SNVer to assess the robustness of SNP and indel detection. Coordinate-level overlap rates were 65–97% for SNPs and 45–87% for indels. Pearson correlations between GATK-derived discrete genotype-frequency estimates and SNVer-derived continuous allele-frequency estimates were highly significant for all pooled datasets (*P* < 10^−300^; Table S3). However, coefficients of determination (*R*^2^) varied among datasets, as expected when discrete genotype representations are compared with continuous allele-frequency spectra derived from pooled, multi-individual samples. Indels exhibited lower overlap rates and *R*^2^ values than SNPs, consistent with the greater susceptibility of short-read indel calls to alignment and representation ambiguity. Second, ADMIXTURE and PCA analyses were repeated after excluding pooled-mite datasets R1–R3 and B1–B19 to assess whether their inclusion influenced the inferred population structure. The broad host-associated pattern was retained. In the ADMIXTURE sensitivity analysis, datasets A15 (*Lynx lynx*; Eurasian lynx) and A35 (*Naemorhedus goral*; Himalayan goral) changed cluster assignment relative to the primary analysis, and the South American camelid-associated datasets resolved into three ancestry subclusters corresponding to the substructure observed in the phylogenetic tree (Fig. 1C). In the PCA sensitivity analysis, A15 remained close to the K6 cluster, and the camelid-associated datasets remained grouped, whereas A35 separated from the K2 cluster, representing the only clear difference from the primary PCA pattern (Table S4; Fig. S1). Together, the primary and sensitivity analyses support a consistent genome-wide pattern of host-associated population structure.

### 3.3 Gene-level variation identifies conserved and highly variable functional gene sets

To characterise how genomic variation is distributed across functional regions, gene-level analyses were conducted on annotated protein-coding genes. Of approximately 9200–9300 predicted protein-coding genes, 5548 CDS and 5264 gene models including introns met coverage criteria and were retained following exclusion of multicopy genes. For CDS, the upper and lower deciles of the nucleotide-diversity distribution each contained 554 genes; for full gene models, including introns, each decile contained 526 genes (Table 3; Fig. 2A). Inclusion of intronic regions resulted in higher overall diversity than for CDS alone. Detailed gene-level nucleotide-diversity estimates for CDS and full gene models are provided in Table S2.

**Table 3:** Summary of gene-level nucleotide diversity (*π*) in *Sarcoptes scabiei*; CDS denotes coding sequences, and Genes include intronic regions; Variable and Invariable correspond to the upper and lower 10% of the *π* distribution, respectively, with remaining genes classified as Neutral; percentages indicate the proportion of genes in each category.

Context	Genes analysed	Variable No. (%)	Neutral No. (%)	Invariable No. (%)	Mean *π*	Min *π*	Max *π*	Mean length (bp)
CDS (exonic)	5548	554 (10.0)	4440 (80.0)	554 (10.0)	0.0029	0.0000	0.0297	1534
Genes (incl. Introns)	5264	526 (10.0)	4212 (80.0)	526 (10.0)	0.0031	0.0000	0.0306	2893

**Fig. 2:**
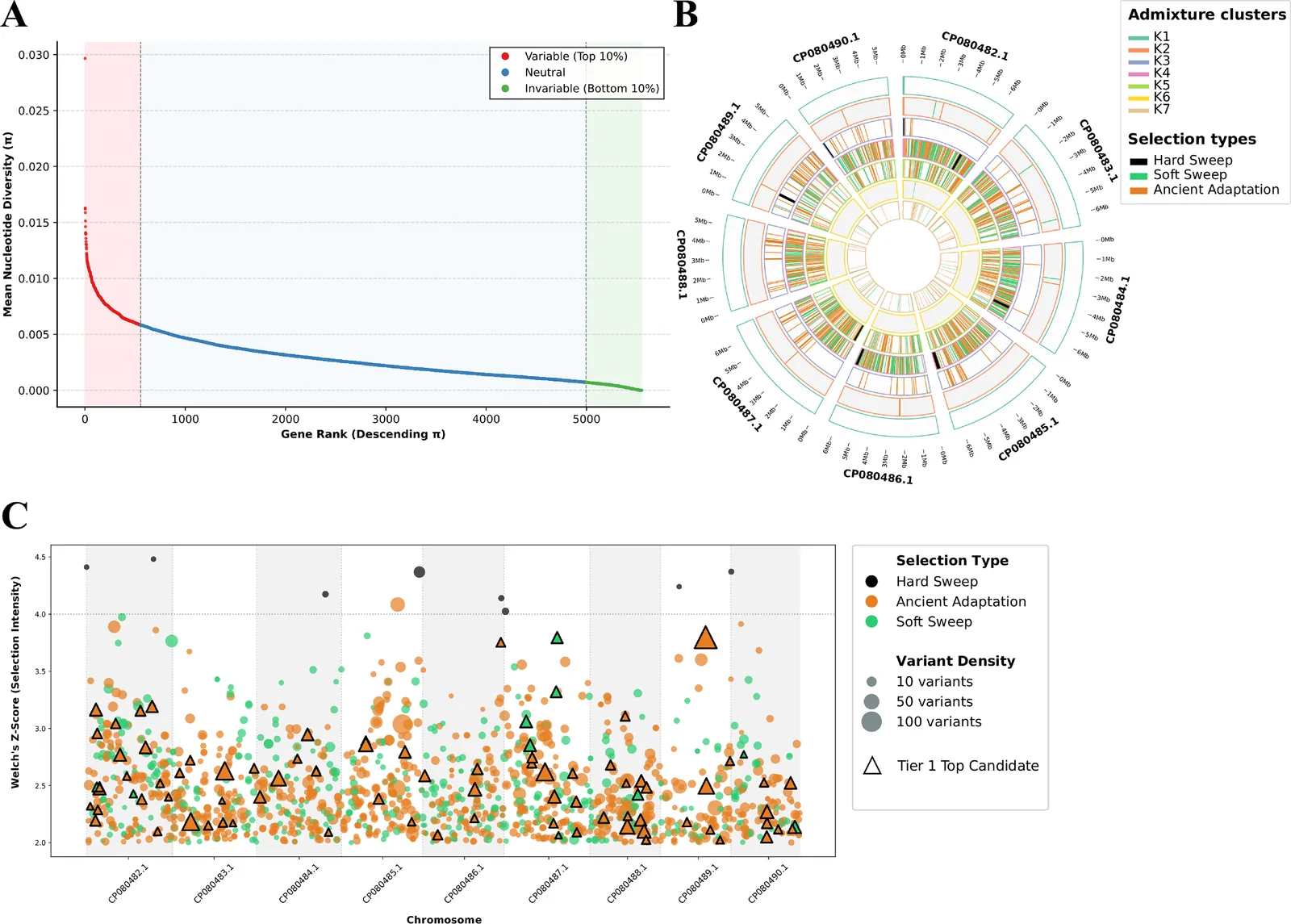
Genome-wide and gene-level variation and candidate signatures of selection in *Sarcoptes scabiei*. (A) Gene-level nucleotide diversity (*π*) ranked across annotated protein-coding genes. Genes are classified as variable (upper 10% of the *π* distribution), invariable (lower 10%) or neutral (remaining genes). (B) Circos plot showing the genomic distribution of final candidate selection blocks across the nine nuclear chromosomes [15]. Nested tracks represent ADMIXTURE-defined clusters (K2–K7; no candidate selection blocks were identified for K1). Final blocks were derived from non-overlapping 10 kb candidate windows evaluated against corresponding 50 kb genomic contexts; adjacent retained windows were merged before classification. Block classifications are indicated as candidate hard-sweep blocks (black), candidate ancient-adaptation blocks (orange) and candidate soft-sweep blocks (green). (C) Manhattan plot of candidate selection signals across chromosomes, showing Welch's *Z*-scores (y-axis) plotted against genomic position (x-axis). Points are coloured by candidate selection class (hard sweep, black; ancient adaptation, orange; soft sweep, green). Symbol size reflects variant density within candidate selection blocks (10, 50 or 100 variants), and triangle symbols denote Tier 1 candidate genes.

Functional annotation indicated that variable genes were associated with categories including DNA repair, cell adhesion and signalling, whereas invariable genes were associated with ribosomal function, oxidative phosphorylation and mitochondrial processes (Table S5). These results describe the distribution of genomic variation across functional gene classes. These gene-level patterns were considered alongside the genome-wide distribution of candidate selection signals (Fig. 2B and C), which is described further in section 3.5.

### 3.4 Genetic differentiation among host-associated populations

The population structure of *S. scabiei* observed across clustering, phylogenetic and multivariate analyses is reflected in genome-wide estimates of genetic differentiation among host-associated clusters (Fig. 3). Pairwise *F*_ST_ estimates ranged from 0.22 to 0.92, with the highest estimate observed between the lynx-associated (K4) and wild rabbit-associated (K5) *S. scabiei* populations, and the lowest between the combined human/pig-associated K1 cluster and the cat/dog-associated K6 cluster.

**Fig. 3:**
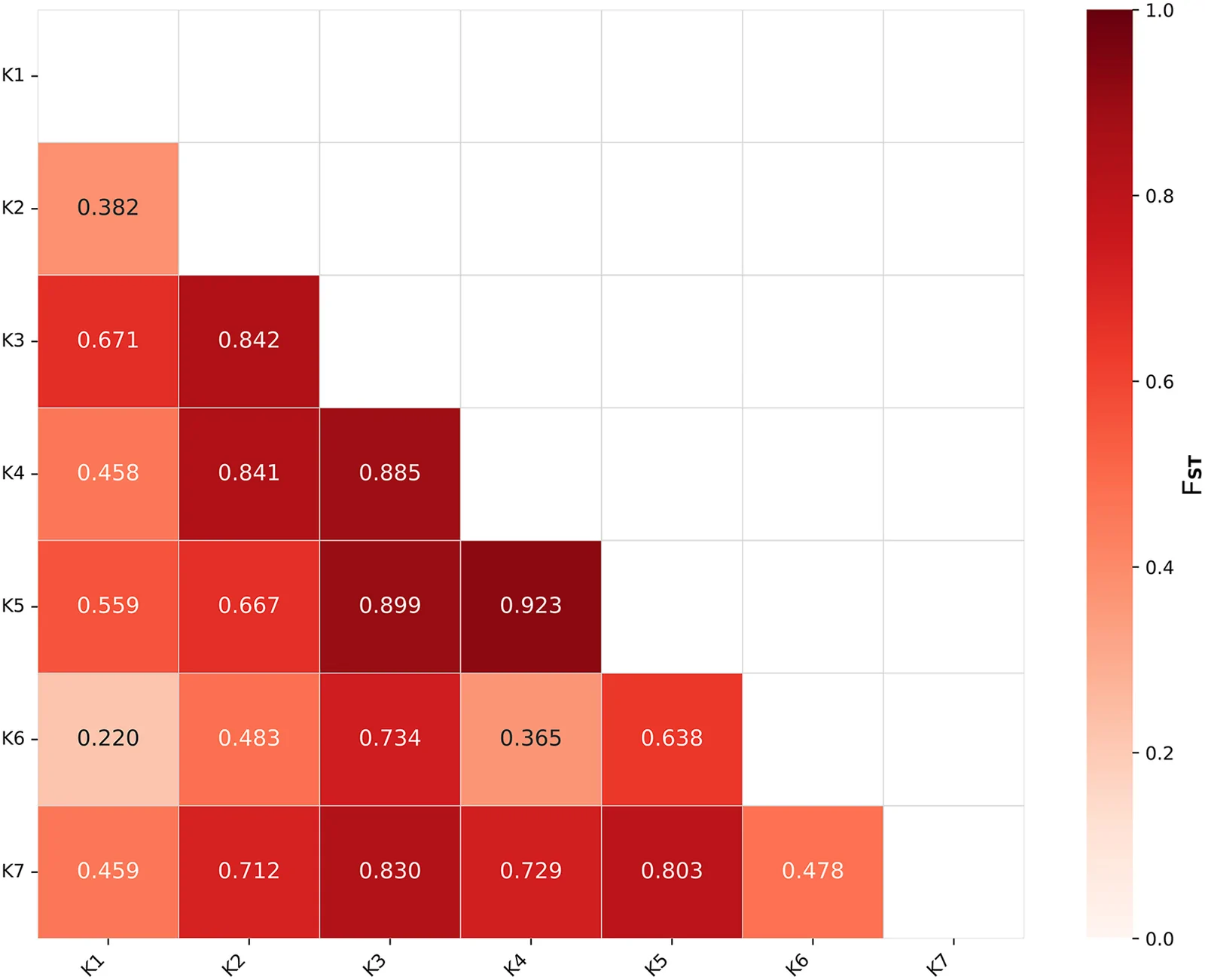
Genetic differentiation among *Sarcoptes scabiei* populations. Heatmap of pairwise fixation-index (*F*_ST_) estimates among ADMIXTURE-defined clusters (K1–K7). Cell values and colour intensity indicate the magnitude of genetic differentiation, with higher estimates representing greater differentiation between host-associated clusters. Pairwise *F*_ST_ estimates were calculated using pixy from filter-passing single-nucleotide polymorphisms (SNPs) within the corresponding 50 kb genomic windows.

The comparison between the domesticated rabbit (K3) and wild rabbit (K5) *S. scabiei* populations also showed high differentiation (*F*_ST_ ≈ 0.90; Fig. 3).

### 3.5 Genome-wide candidate selection signals and distribution of candidate genes across host-associated clusters

Building on these patterns of genome-wide and gene-level variation, selection analyses identified 1600 candidate selection blocks across the ADMIXTURE-defined clusters, comprising eight candidate hard-sweep blocks, 486 candidate soft-sweep blocks and 1106 candidate ancient-adaptation blocks; six additional blocks were classified as conserved low-diversity blocks (Fig. 2B and C; Table S6). These candidate selection blocks contained 46,545 cluster-specific variant records with ΔAF > 0.8, comprising 28,794 SNP records, 384 MNP records and 17,367 indel records (Table S7). Collectively, the candidate selection and conserved low-diversity blocks overlapped 3742 unique protein-coding genes, represented by 5782 gene–cluster records (Table S8). Candidate genes were ranked within each cluster using the composite selection metrics described in the Methods.

Three genes (evm.model.Hic_asm_8.879, evm.model.Hic_asm_8.378 and evm.model.Hic_asm_8.379) overlapped conserved low-diversity blocks characterised by minimal variation across all clusters (Table S8). Of the 5782 gene–cluster records, 780, representing 681 unique genes, were classified as Tier 1. A total of 157 variant records associated with Tier 1 genes met the predefined high-confidence criteria based on allele-frequency differentiation and genomic context (Tables S7 and S8). Top-ranked candidate genes are summarised in Table 4. Functional enrichment analysis of Tier 1 genes identified KEGG pathways and BRITE categories that were nominally enriched in host-associated clusters K3, K4, K5 and K7 (Table S9).

**Table 4:** Representative candidate genes and associated high-confidence variants in candidate selection blocks of *Sarcoptes scabiei* (ΔAF > 0.8). Clust. denotes the assigned ADMIXTURE cluster (K3, K4, K5 or K7); Block class indicates the candidate selection classification (Anc. = ancient adaptation; Hard = hard sweep; Soft = soft sweep); Var. is the number of high-confidence variants per gene. R_C_ is the maximum variant-level composite ranking score, calculated as ΔAF × *Z*, among the high-confidence variants associated with each gene.

Gene identification	Clust.	Block class	Var.	R_C_	Function
evm.model.Hic_asm_7.420	K3	Anc.	2	3.798	Hypothetical protein
evm.model.Hic_asm_7.614.1	K3	Anc.	1	2.715	T-box transcription factor
evm.model.Hic_asm_1.819	K3	Anc.	1	2.629	Hypothetical protein
evm.model.Hic_asm_5.764	K5	Soft	1	3.635	Disorder prediction
evm.model.Hic_asm_5.742	K5	Soft	2	3.204	Disorder prediction
evm.model.Hic_asm_0.929	K5	Anc.	1	3.193	BAR domain
evm.model.Hic_asm_4.1040	K4	Anc.	1	3.627	Hypothetical protein
evm.model.Hic_asm_0.765	K4	Anc.	1	3.135	Disorder prediction
evm.model.Hic_asm_0.164	K4	Anc.	4	3.144	ThDP-binding fold
evm.model.Hic_asm_1.289	K7	Anc.	1	2.722	Cyclin B1
evm.model.Hic_asm_1.919	K7	Anc.	2	2.152	Hypothetical protein

*Abbreviations:* BAR, Bin/Amphiphysin/Rvs domain; ThDP, thiamin diphosphate; ΔAF, absolute.

allele-frequency difference; Z, standardised selection contrast.

## 4 Discussion

This study provides a genome-wide analysis of nuclear genetic variation in *S. scabiei* across a broad range of host species and geographical regions, showing that host association is a prominent correlate of genetic structure across the sampled populations. Using 83 quality-filtered whole-genome datasets, ADMIXTURE, principal component and phylogenetic analyses identified seven ancestry clusters broadly corresponding to host-associated groups, with pairwise *F*_ST_ values ranging from 0.22 to 0.92. These findings reinforce earlier mitochondrial and microsatellite-based studies [[8], [9], [10],^38^] and extend the chromosome-scale findings of Xu et al. [15] through broader multi-host sampling and integrated analyses of ancestry, phylogenetic relationships, genome-wide diversity and differentiation, gene-level variation and candidate selection signals. The present work therefore broadens the population-genomic evidence for host-associated structuring in *S. scabiei*. Across the approximately 56 Mb nuclear genome, 698,136 high-quality SNPs were identified, providing a substantial resource for investigating genome-wide variation in this parasite.

Genome-wide nucleotide diversity estimates varied markedly among ancestry clusters (Table 2). The highest mean values were observed in the combined human/pig-associated K1 cluster (*π* = 0.1945) and the cat/dog-associated K6 cluster (*π* = 0.1537), whereas substantially lower values occurred in the lynx-associated K4 cluster (*π* = 0.0126) and the wild rabbit-associated K5 cluster (*π* = 0.0225). The absolute genome-wide *π* estimates should be interpreted cautiously because the relatively limited number of retained invariant positions may have inflated their magnitude. However, all clusters were analysed using the same filtered set of nucleotide positions; consequently, the relative differences in nucleotide diversity among clusters remain valid and form the basis of the comparative interpretation. Pairwise differentiation was similarly heterogeneous: the highest *F*_ST_ value was observed in *S. scabiei* between the lynx-associated K4 and the wild rabbit-associated K5 clusters, and strong differentiation was also detected in *S. scabiei* between the domesticated rabbit-associated K3 and the wild rabbit-associated K5 clusters. These differences may reflect variation in demographic history, population connectivity, founder effects, host-associated isolation, geographical sampling breadth and composite signals from pooled-mite datasets. Because several clusters contained mites from more than one host species and differed in their geographical representation, the observed diversity patterns cannot be attributed to host species alone. Greater standing genetic variation in some populations could provide greater opportunity for resistance-associated alleles to arise or persist; however, known acaricide-resistance loci were not specifically investigated in this study.

The present findings support strong host-associated structuring but do not exclude occasional cross-host connectivity. They do not, however, establish the frequency, direction or epidemiological importance of such events. Mitochondrial studies have reported occasional clustering of human- and animal-derived mites within shared clades, raising the possibility of connectivity among mites from different hosts [39], whereas population-genetic analyses based on microsatellite and nuclear markers have generally supported host-associated structuring with restricted gene flow [9,10]. The combined human/pig-associated K1 and cat/dog-associated K6 clusters further show that ancestry groups are not invariably restricted to a single host species. Experimental infestation studies also indicate that successful establishment following cross-infestation is influenced by host biology, immune responses and exposure history [40]. Broader and more balanced sampling across host–geography combinations will therefore be required to distinguish host-associated from geographical effects and to resolve contemporary population connectivity and transmission dynamics.

The contrasting relationships between mites from domesticated and wild hosts further illustrate the possible contributions of host history, husbandry and geography. Mites from domesticated rabbits formed a highly divergent and well-supported lineage distinct from those infecting wild rabbits, whereas mites from domesticated pigs and wild boars clustered closely together. The close phylogenetic relationship of *S. scabiei* from domesticated pigs and wild boars may reflect the complex domestication history of pigs, which involved repeated introgression and incomplete genetic separation from wild boar populations [41]. In contrast, rabbit domestication involved marked demographic and genetic change [42]. The divergence in *S. scabiei* between domesticated and wild rabbits may partly reflect differences in host history, husbandry, geography and opportunities for contact between managed and wild populations. These factors may have restricted parasite gene flow and promoted divergence of the associated *S. scabiei* lineages. Nevertheless, the potential influence of geographical sampling bias cannot be excluded.

The multi-metric analysis identified 1600 candidate selection blocks, comprising eight candidate hard-sweep blocks, 486 candidate soft-sweep blocks and 1106 candidate ancient-adaptation blocks, together with six conserved low-diversity blocks (Fig. 2B and C; Table S6). These categories are operational genomic classifications consistent with selection acting over different evolutionary timescales; they do not represent experimentally established evolutionary histories. Candidate hard-sweep blocks may reflect relatively recent allele-frequency shifts, whereas candidate soft-sweep blocks are consistent with selection acting on standing variation. Candidate ancient-adaptation blocks combined elevated relative differentiation and absolute divergence and may therefore reflect longer-term population separation. Candidate ancient-adaptation blocks were the most numerous, whereas candidate hard-sweep blocks were comparatively rare. The candidate selection blocks contained 46,545 cluster-specific variant records with ΔAF > 0.8, comprising 28,794 SNP records, 384 MNP records and 17,367 indel records; 157 variant records met the predefined high-confidence criteria (Table S7). These genomic signals identify candidate blocks and variants whose phenotypic consequences remain to be established.

Gene-level analyses provided complementary evidence of heterogeneous functional constraint. Variable gene sets showed nominal enrichment for DNA repair processes, particularly homologous recombination, as well as cell adhesion and signalling, suggesting that variation in these functions may contribute to biological differences among the sampled populations (Table S5). Enrichment of homologous–recombination pathways may reflect variation in genome–maintenance processes rather than direct evidence of host adaptation. In contrast, invariable gene sets showed nominal enrichment for essential cellular processes, including ribosomal function, oxidative phosphorylation and mitochondrial biogenesis, consistent with functional constraint on core biological functions. Additional nominal enrichment of ubiquitin-mediated protein degradation and secretion systems suggests that protein turnover and export machinery are highly conserved across the sampled populations. Functional-enrichment profiles also differed among the K3, K4, K5 and K7 clusters, although these associations do not by themselves demonstrate functional adaptation to particular hosts (Table S9). Representative Tier 1 candidates included evm.model.Hic_asm_7.614.1, annotated as a T-box transcription factor; evm.model.Hic_asm_0.929, containing an Arfaptin homology/Bin/Amphiphysin/Rvs domain; evm.model.Hic_asm_0.164, containing a thiamin diphosphate-binding fold; and evm.model.Hic_asm_1.289, annotated as G2/mitotic-specific cyclin B1 (Table 4; Table S8). In the respective K3, K5, K4 and K7 analyses, each gene occurred within a candidate ancient-adaptation block and contained at least one putative exonic frameshift indel (Table S8). These candidates span regulatory, membrane-associated, metabolic and cell-cycle-related functions, although their roles in host association or mite biology require experimental validation. Other candidate categories included Toll/interleukin-1 receptor-domain-containing proteins, proteasome components and RNA-binding proteins, while several highly ranked genes remained annotated as hypothetical proteins (Table S8). Given that cellular immune mechanisms and parasite immune–evasion processes influence scabies outcomes [40,[43], [44], [45]], variation in signalling and proteolytic functions, as well as in functions putatively involved in immune interactions, is biologically plausible. Nevertheless, functional enrichment and genomic co-location alone do not establish that the corresponding genes mediate host adaptation, immune evasion or pathogenicity.

Several limitations should be considered. Host species and geographical origins were represented unevenly, and relatively few human-derived datasets were retained, limiting the extent to which host-associated and regional effects could be separated. Some host groups included pooled-mite datasets, for which allele-frequency and heterozygosity estimates represent composite sample-level signals rather than individual-mite genotypes. Accordingly, frequency-based statistics that incorporated pooled datasets were interpreted as comparative sample- or cluster-level estimates rather than as direct measurements of allele frequencies in individual mites. Cross-validation against SNVer supported the robustness of site-level variant detection in pooled samples, but standard diploid genotype calls and continuous pooled allele-frequency estimates are not directly equivalent (Table S3). Sensitivity analyses excluding pooled-mite datasets largely preserved the principal ADMIXTURE and PCA patterns, indicating that the broad host-associated population structure was not driven by the inclusion of pooled material and supporting retention of these datasets in the primary analyses (Table S4; Fig. S1). Sequencing depth varied substantially among datasets, although conservative site- and sample-level filters were applied to reduce coverage-related artefacts.

Quality filtering excluded 42 of the 125 initial sample-level datasets, predominantly because of high missingness and/or heterozygosity (Table S1). The underlying causes could not be established with certainty. Low-input or degraded field-derived DNA, variable sequencing depth, residual host-derived sequence content and mapping uncertainty may have contributed to these outcomes in some datasets, while genuine within-sample variation may also elevate heterozygosity, particularly in pooled material. Conservative filtering was used to minimise downstream artefacts, although the exclusions reduced the representation of some host and geographical groups. In addition, genome-wide and gene-level nucleotide-diversity estimates were calculated using different denominators and should not be interpreted as directly equivalent. Finally, the inferred selection classes, differentiated variants and ranked genes represent candidate genomic signals rather than experimentally validated mechanisms of adaptation.

The strong host-associated structure observed here does not weaken the rationale for a One Health approach; rather, it supports an integrated but host-stratified framework. Human scabies control, companion-animal treatment, livestock management and wildlife surveillance are likely to require different interventions because population structure differs among host groups and transmission dynamics may also be host-specific. Nevertheless, coordinated genomic surveillance, harmonised metadata and communication among human, veterinary and wildlife health sectors could help distinguish sustained transmission within host-associated populations from possible cross-host spillover. The present dataset also provides a basis for targeted investigation of resistance-associated loci, including the voltage-gated sodium channel locus, although acaricide resistance was not directly assessed here [46]. Similarly, the conserved and variable genes identified in this study provide starting points for future therapeutic, diagnostic and vaccine research, although their translational relevance requires validation.

## 5 Conclusions

Genome-wide analysis of *S. scabiei* reinforces and extends evidence of strong host-associated population structure and identifies heterogeneous patterns of diversity, differentiation and candidate selection signals across the sampled populations. These findings provide a population-genomic foundation for investigating the evolution and epidemiology of this parasite and for informing future host-stratified One Health surveillance of scabies and sarcoptic mange.

## CRediT authorship contribution statement

**Fang Fang:** Writing – review & editing, Writing – original draft, Supervision, Resources, Project administration, Investigation, Funding acquisition, Formal analysis, Data curation, Conceptualization. **Pasi K. Korhonen:** Writing – review & editing, Validation, Software, Resources, Methodology, Investigation, Formal analysis, Data curation. **Siyi Huang:** Writing – review & editing, Validation, Resources, Investigation. **Barbara Moroni:** Writing – review & editing, Resources, Investigation. **Luca Rossi:** Writing – review & editing, Validation, Supervision, Resources, Investigation, Conceptualization. **Anna R. Molinar Min:** Writing – review & editing, Validation, Resources, Investigation. **Zhijun Hou:** Writing – review & editing, Resources, Investigation. **Vito Colella:** Writing – review & editing, Resources. **Qingyou Liu:** Writing – review & editing, Resources, Investigation. **Jacques Guillot:** Writing – review & editing, Supervision, Resources, Investigation, Data curation. **Robin B. Gasser:** Writing – review & editing, Writing – original draft, Supervision, Resources, Project administration, Methodology, Investigation, Funding acquisition, Data curation, Conceptualization.

## Ethics declaration

This study was conducted in accordance with the following guidelines for animal welfare and/or reporting: Guangxi University Animal Ethics Committee. This study was approved by the Guangxi University Ethics Committee. (Approval No. GXU-2023-0040)

## Consent for publication

Not applicable.

## Availability of data and materials

Whole-genome sequence data and associated metadata generated in this study have been deposited in the NCBI Sequence Read Archive under BioProject accession number PRJNA1467743. Metadata include host species, geographical origin and sample type. Accession numbers for individual sequencing datasets are SRR38727674–SRR38727843.

## Declaration of generative AI and AI-assisted technologies in the manuscript preparation process

During the preparation of this work, the authors used ChatGPT (OpenAI) to assist with the editing and formatting of some sections. After using this tool, the authors reviewed and edited the content and take responsibility for the content of the published article.

## Funding

This work was supported by the 10.13039/501100001809National Natural Science Foundation of China [grant number 31902293] and the 10.13039/501100000923Australian Research Council [grant codes LP180101085 and LP220200614].

## Declaration of competing interest

The authors declare that they have no known competing financial interests or personal relationships that could have appeared to influence the work reported in this paper.

## Figures and Tables

**Fig. S1:**
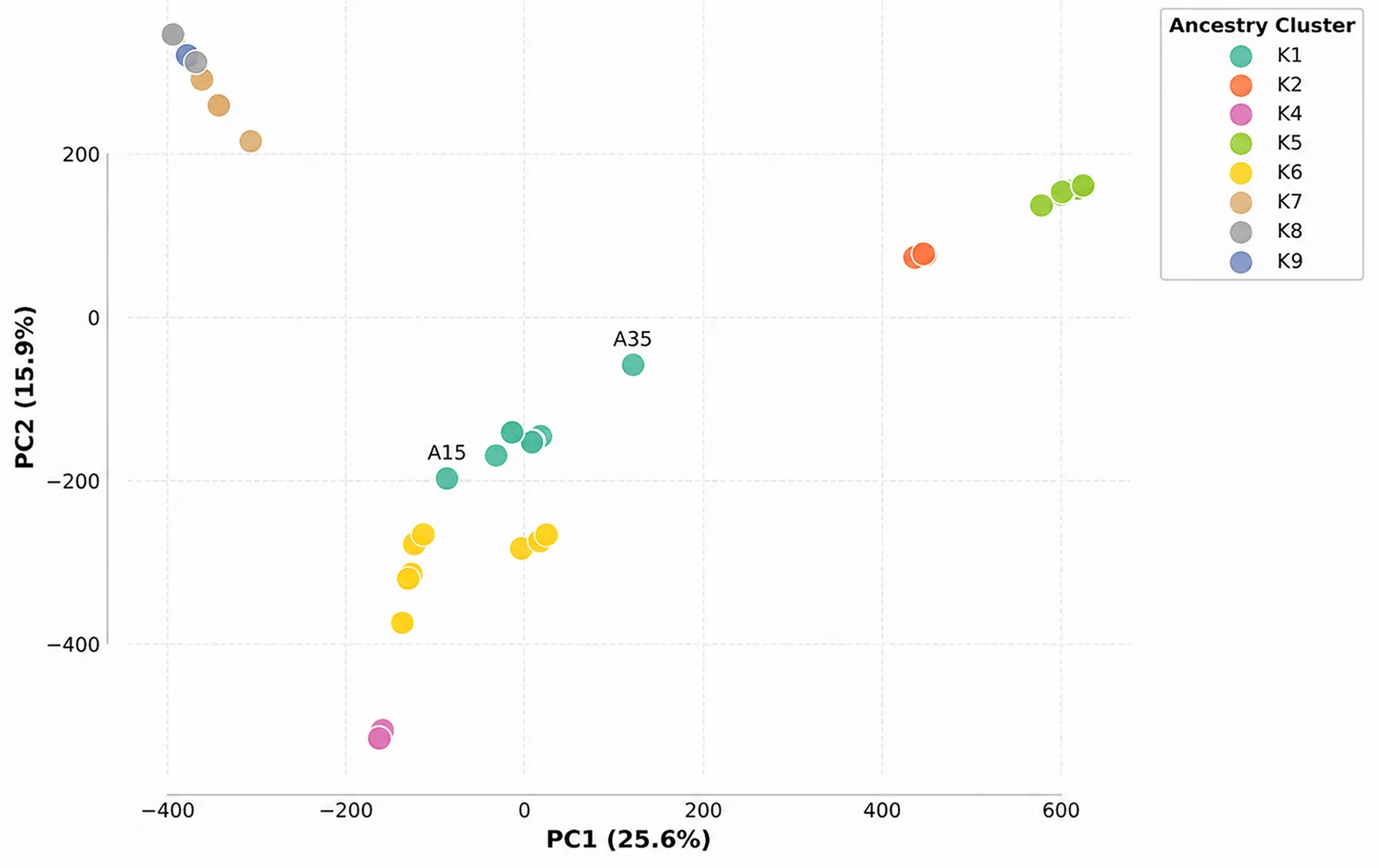
Principal component analysis (PCA) after exclusion of pooled-mite datasets. PCA was performed after excluding pooled-mite datasets R1–R3 and B1–B19. Each point represents an individual-mite dataset and is coloured according to its cluster assignment in the corresponding ADMIXTURE sensitivity analysis (Table S4). The broad host-associated grouping observed in the primary analysis was retained. South American camelid-associated datasets assigned to clusters K7–K9 remained grouped in the PCA. Dataset A15 (*Lynx lynx*; Eurasian lynx), which changed ADMIXTURE cluster assignment after exclusion of the pooled datasets, remained close to the K6 cluster. In contrast, dataset A35 (*Naemorhedus goral*; Himalayan goral) separated from the K2 cluster to which it was assigned in the primary analysis.
